# Characterization of knee alignment in children with mucopolysaccharidosis types I and II and outcome of treatment with guided growth

**DOI:** 10.1007/s11832-015-0661-0

**Published:** 2015-06-16

**Authors:** Elizabeth Ashby, Deborah Eastwood

**Affiliations:** Department of Orthopaedic Surgery, Great Ormond Street Hospital for Children, Great Ormond Street, London, WC1N 3JH UK

**Keywords:** Mucopolysaccharidoses, Knee alignment, Guided growth

## Abstract

**Purpose:**

To describe knee alignment in children of different ages with severe mucopolysaccharidosis (MPS) I and II and the outcome of treatment with guided growth in a patient subgroup.

**Methods:**

This is a retrospective observational study of 58 knees in 29 children with severe MPS I and II. Long-leg standing radiographs were evaluated to determine mechanical axis deviation, mechanical lateral distal femoral angle and medial proximal tibial angle at different ages throughout childhood. The change in deformity in individual children over time is reported. 20 knees in 10 patients were treated with guided growth using eight-plates. Radiographic measurements were recorded at the time of plate insertion, at plate removal and at 1 year following removal.

**Results:**

At 8 years of age, all MPS I children and three-quarters of MPS II children had valgus knee alignment. There was deformity progression in two-thirds of MPS I knees and half of MPS II knees. Guided growth corrected the deformities. There was recurrence in most cases 1 year after plate removal.

**Conclusions:**

Knee deformity is common in children with severe MPS I and II. Guided growth can be considered where there is significant and/or or progressive deformity with the aim of halting progression and correcting existing deformity and thus minimizing the risk of gross deformity. Patients should be aware of the high rate of recurrence and the need for repeat surgery.

## Introduction

The mucopolysaccharidoses (MPS) are a group of lysosomal storage diseases characterized by the absence, reduction or malfunctioning of a lysosomal enzyme. Cellular glycosaminoglycans (GAGs) accumulate and lead to tissue and multi-organ dysfunction. There are different types of MPS based on the dysfunctional enzyme. This study focuses on MPS I and II.

The prevalence of MPS I and II is approximately 1 in 100,000 [1, 2]. MPS I is an autosomal recessive disorder characterized by dysfunctional α-L-iduronidase leading to accumulation of dermatan sulphate and heparan sulphate. MPS I is recognized as a spectrum of disorders that are split into a ‘severe’ and ‘attenuated’ form; however, they are biochemically identical.

MPS II is an X-linked recessive disorder characterized by dysfunctional iduronate sulphatase leading to accumulation of the same GAGs as in MPS I. MPS II encompasses a spectrum of disorders that are divided into 2 broad subtypes—a severe form characterized by the presence of cognitive impairment and a less severe form with no cognitive impairment.

Infants with MPS I and II typically have normal development for the first 6−12 months of life [3]. Without treatment, children with severe MPS I usually die in their first decade [4]. Children with attenuated MPS I have a relatively normal life expectancy, but with significant morbidity [4]. Without treatment, children with severe MPS II typically die in the second decade of life [5]. Those with the less severe form can survive until their fifth or sixth decade [4].

A range of skeletal radiographic abnormalities known as ‘dysostosis multiplex’ are associated with MPS I [4] including hypoplastic vertebral bodies (resulting in kyphosis and scoliosis), poor growth of long bones, hip dysplasia and genu valgum [6, 7]. The rate of genu valgum in MPS I is between 52 [8] and 70 % [9]. The deformity is thought to originate from failure of ossification of the lateral margin of the proximal tibial metaphysis [6]. ‘Dysostosis multiplex’ is less dramatic in MPS II [10]. The incidence of genu valgum is unknown.

Haematopoietic stem cell transplantation (HSCT) is used to treat severe MPS I [11]. Donor cells synthesise a continuous endogenous supply of the deficient enzyme leading to increased life expectancy and improved cognitive and physical function. HCST has a mortality rate of 15 % [12]. It has been used to treat MPS II [13] but is not usual practice. HCST has little effect on the musculoskeletal system [14]. Odontoid hypoplasia shows some improvement [15] but hip dysplasia and genu valgum continue to progress [16, 17]. This may be due to poor vascularity of the ground substance of musculoskeletal tissues precluding enzyme access.

Enzyme replacement therapy (ERT) is used to treat attenuated MPS I and severe and attenuated MPS II. In attenuated MPS I, recombinant human α-L-iduronidase results in short- and medium-term clinical improvements [18]. Enzymes are unable to cross the blood–brain barrier so provide no cognitive benefit in severe MPS I. Recombinant human iduronate-2-sulfatase is beneficial in severe [19] and attenuated MPS II [20]. Although ERT can improve joint range of motion, no improvement is seen in hip dysplasia or genu valgum [21].

Improved life expectancy and quality of life, resulting from HSCT and ERT, have resulted in more patients and doctors considering surgical intervention for genu valgum. Surgical correction of knee deformity using guided growth was first reported using staples [22]. More recently, the use of eight-plates has been described [8] but timing, indications and recurrence rates remain unclear.

The aims of this retrospective observational study are three-fold. Firstly, to describe knee deformity in children of different ages with severe MPS I and II. Secondly, to describe deformity progression in individual children, and thirdly, to report the outcome of treatment with guided growth in a subgroup of patients who demonstrated progressive deformity.

## Materials and Methodology

All patients with MPS I or II, who were treated at our institution between January 2004 and December 2014, with a minimum of one full-length standing leg X-ray were included in the study. X-rays were standardised with patellae facing forward, a level pelvis and a measurement ball on each film.

A total of 29 children (58 legs) were included in the study—17 children with severe MPS I and 12 children with severe MPS II. There were 10 males and 7 females in the MPS I group. The MPS II group was entirely male. All MPS I patients had received HSCT (2/17 subsequently received ERT). All MPS II patients were receiving ERT. Treatments were started prior to radiographic assessment of knee alignment.

**Fig. 1:**
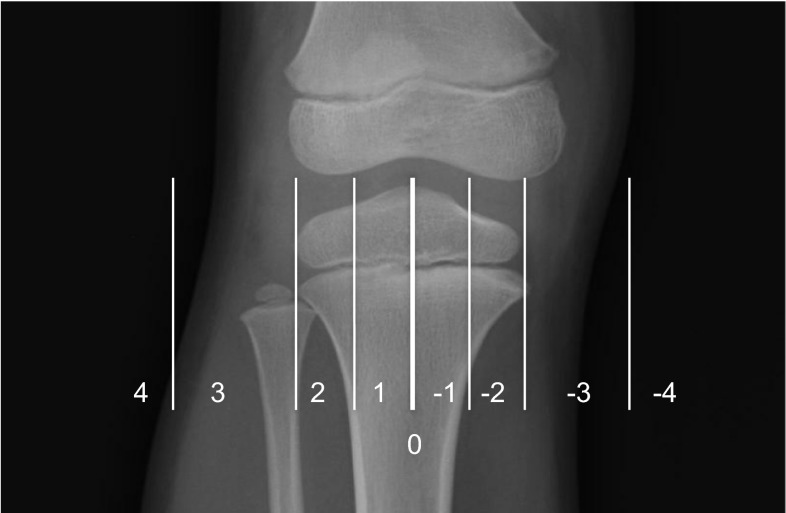
Radiograph showing the zones of the knee. Zone 0 is a* vertical line* through the central point of the tibial plateau (*thick* *white* *line*). Each half of the tibial plateau is divided into two. Zone 1 is located next to the central line and zone 2 is more peripheral. A* vertical line* is drawn on either side of the knee at a distance equal to the width of the tibial hemi-plateau. Zone 3 lies between the tibial plateau and this line and zone 4 lies outside this line. The mechanical axis deviation zones have positive values in a valgus knee and negative values in a varus knee

The Picture Archiving and Communication System was used to determine the alignment of each knee by measuring mechanical axis deviation (MAD). MAD was classified as passing through one of four zones. A positive value indicated a valgus knee and a negative value indicated a varus knee. Zones were calculated in relation to the width of the tibial plateau (Fig. 1). The source of knee deformity was determined by measuring the mechanical lateral distal femoral angle (mLDFA) and the medial proximal tibial angle (MPTA).

The mean and range of each radiographic measure is reported for X-rays between the ages of 2 and 9 years for MPS I and between the ages of 4 and 13 years for MPS II (or within 6 months of each time point). No child with MPS I had a limb alignment X-ray performed under the age of 2 years and no child with MPS II had an X-ray performed under the age of 4 years. Corrective surgery was performed at a mean age of 9 years in MPS I and 13 years in MPS II. Radiographs after the implementation of guided growth techniques are not reported in this part of the study as they would not be representative of natural progression. In total, 67 radiographs (134 knees) in children with MPS I and 37 radiographs (74 knees) in children with MPS II were evaluated.

To assess disease progression in individual children, only those with a minimum of 3 X-rays prior to any surgical intervention were included for analysis. There were 12 children with MPS I (24 knees) and 8 children with MPS II (16 knees). Details of the number and timing of X-rays are shown in Table 1. Radiographic findings on the initial X-ray were compared to those on the final X-ray. To assess the rate of disease progression, the change in radiographic appearance was divided by the time taken for the change to occur.

**Table 1:** Number and timing of full-length leg X-rays used to assess knee alignment over time

	MPS I	MPS II
Number of knees evaluated	24	16
Mean number of knee X-rays taken (range)	4.8 (3−12)	3.6 (3−5)
Mean age at first X-ray (range)	6.9 years (2.3–10.4)	7.5 years (4.2–13.8)
Mean age at final X-ray (range)	10.0 years (5.2–13.0)	11.2 years (5.8–16.2)
Mean time between first and final X-ray (range)	3.1 years (1.0–4.9)	3.7 years (0.9–5.1)

A total of 10 children (7 with MPS I and 3 with MPS II) were treated with guided growth using Orthofix eight-plates. Indications for surgery included a MAD through zone 3 or higher or a MAD through zone 2 together with evidence of deformity progression. The origin of the knee deformity was established from the mLDFA and MPTA. Eight-plates were inserted into the proximal tibia and/or the distal femur as appropriate. Radiographic measurements at the time of eight-plate insertion, at eight-plate removal and at 1 year following eight-plate removal are reported.

## Results

### Knee alignment in children of different ages

**Fig. 2:**
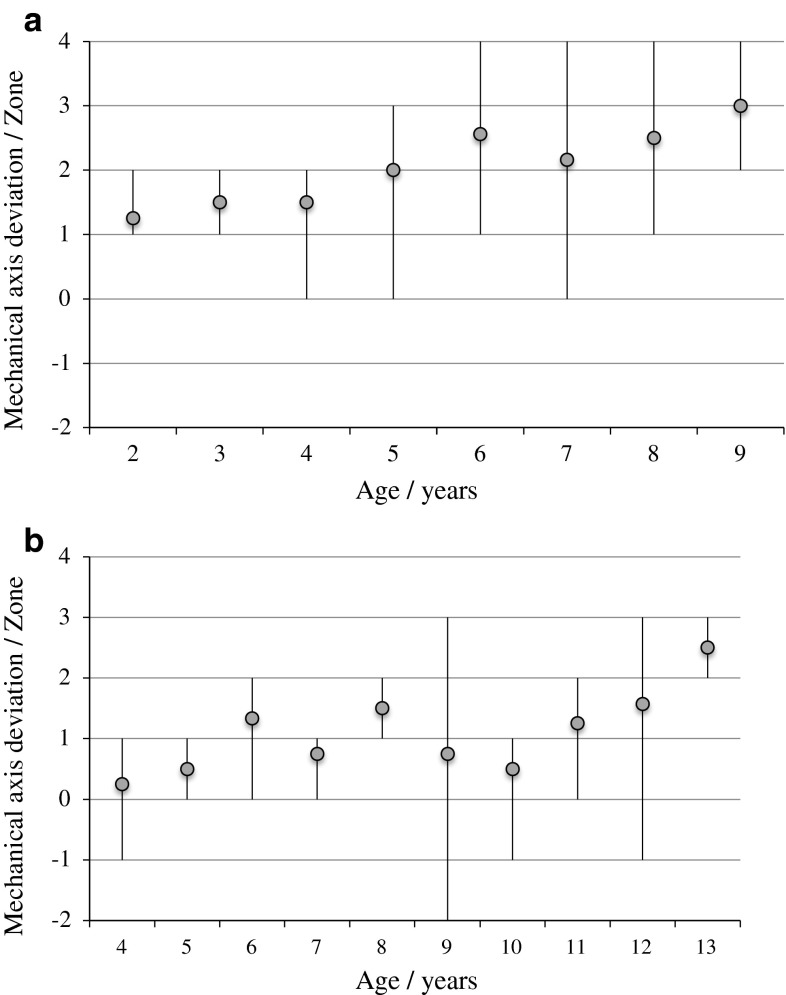
**a** Mechanical axis deviation in children with MPS I at different ages. *Grey* *dot* represents the mean value and the *black* *line* represents the range. **b** Mechanical axis deviation in children with MPS II at different ages. *Grey* *dot* represents the mean value and the *black* *line* represents the range

**Fig. 3:**
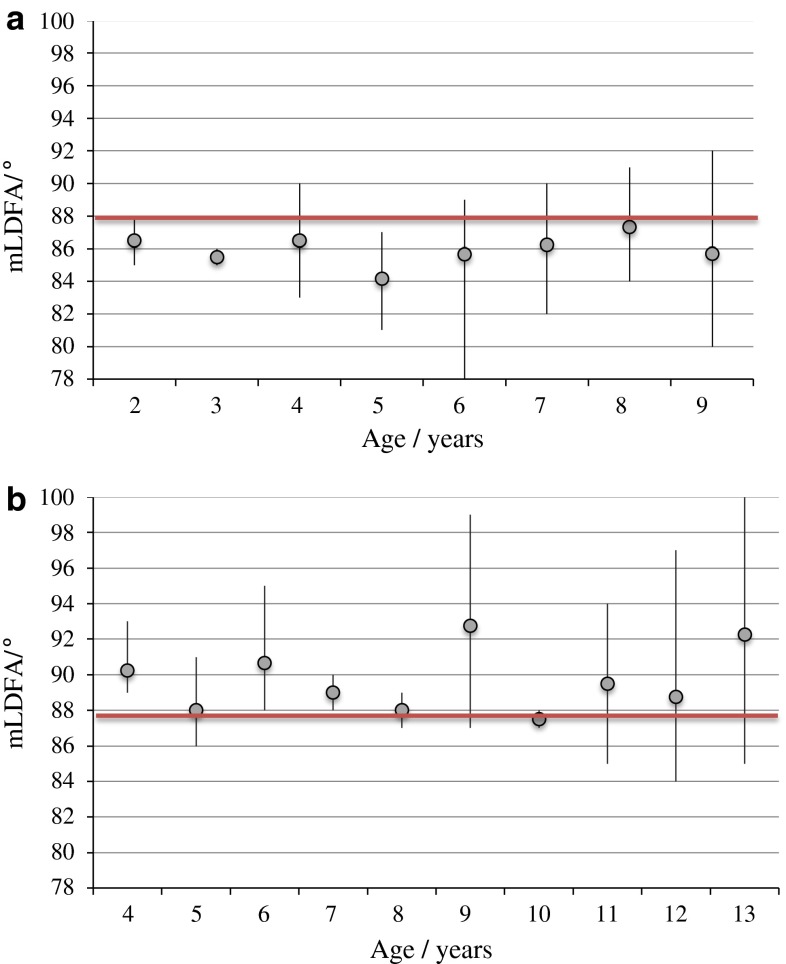
**a** Mechanical lateral distal femoral angle (mLDFA) in children with MPS I at different ages. *Grey* *dot* represents the mean value and the *black* *line* represents the range. The heavy *horizontal* *line* highlights the normal value of 88°. **b** Mechanical lateral distal femoral angle (mLDFA) in children with MPS II at different ages. *Grey* *dot* represents the mean value and the *black* *line* represents the range. The heavy *horizontal* *line* represents the normal value of 88°

**Fig. 4:**
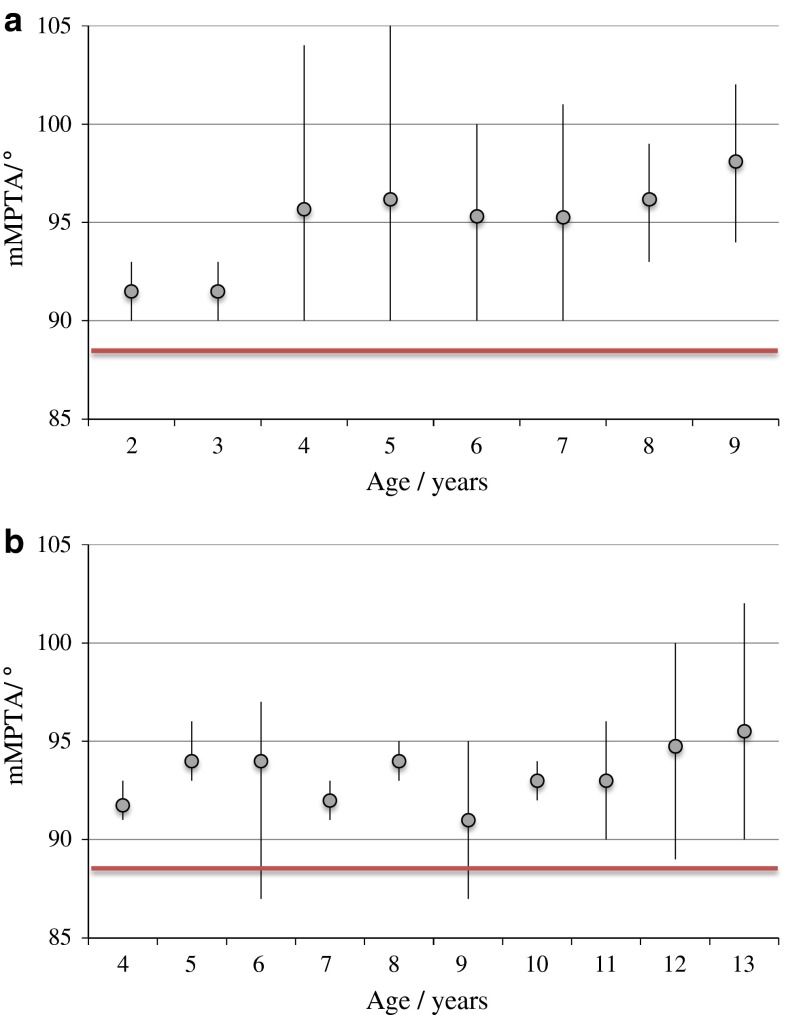
**a** Medial proximal tibial angle (MPTA) in children with MPS I at different ages. *Grey* *dot* represents the mean value and the *black* *line* represents the range. The heavy *horizontal* *line* represents the normal value of 88°. **b** Medial proximal tibial angle (MPTA) in children with MPS II at different ages. *Grey* *dot* represents the mean value and the *black* *line* represents the range. The heavy *horizontal* *line* represents the normal value of 88°

The MAD, mLDFA and MPTA were assessed in 29 children (58 legs) at different ages. Results are shown in Figs. 2, 3 and 4, respectively, highlighting the differences between MPS I and II patients.

### Deformity progression in individual children

Deformity progression over time was assessed in 20 children (40 knees). Results are shown in Table 2. In knees with progressive deterioration in mechanical axis deviation, the mean rate of change in MPS I was 0.51 zones/year (range 0.20–1.10) and in MPS II was 0.59 zones/year (range 0.33–1.54).

**Table 2:** Change in mechanical axis deviation in individual children with time

Change in MAD with time	MPS I	MPS II
Deteriorates	15/24 (63 %)	8/16 (50 %)
Remains constant	9/24 (37 %)	6/16 (38 %)
Improves	0/24 (0 %)	2/16 (12 %)

### Results of treatment with guided growth

A total of 10 children (20 knees) were treated with guided growth. The plates were removed in 6 cases (12 knees); details are given in Table 3. There was correction of deformity to MAD zone –1, 0 or 1 in all cases. The mean time plates remained in situ was 1.6 years. Deformity recurred in 3 children (6 knees) following plate removal. Correction was maintained in one patient (2 legs) who was skeletally mature at the time of plate removal. Details of the 4 children (8 knees) where eight-plates remained in situ are given in Table 4. The MAD is showing improvement in all cases.

**Table 3:** Details of guided growth procedures performed on 6 children (12 knees) where the plates have been removed

Child number	Type of MPS	Gender	Side of operation	Bone primarily causing deformity	Age eight-plate(s) inserted/years	Age eight-plate(s) removed/years	Time eight-plate in situ/years	MAD Zone before eight-plate insertion	MAD Zone after eight-plate removal	MAD Zone one year after removal
1	I	F	Right	Tibia/femur	6.5	8.0	1.5	4	0	4
			Left	Tibia/femur	6.5	8.0	1.5	4	1	3
2	I	F	Right	Femur	7.3	8.7	1.4	3	–1	3
			Left	Femur	7.3	8.7	1.4	4	1	3
3	I	F	Right	Tibia/femur	9.6	10.7	1.1	3	–1	2
			Left	Tibia/femur	9.6	10.7	1.1	3	0	1
4	II	M	Right	Tibia	11.8	13.5	1.7	2	–1	^a^
			Left	Tibia	11.8	13.5	1.7	3	1	^a^
5	I	M	Right	Tibia	10.7	12.4	1.7	2	0	^a^
			Left	Tibia	10.7	12.4	1.7	4	1	^a^
6	II	M	Right	Tibia	14.2	16.2	2.0	2	0	0
			Left	Tibia	14.2	16.2	2.0	3	1	1

^a^Insufficient time elapsed to provide figures for patients 4 and 5

**Table 4:** Details of guided growth procedures performed on 4 children (8 knees) where the plates remain in situ

Child number	Type of MPS	Gender	Side of operation	Bone primarily causing deformity	Age eight-plate(s) inserted/years	Time since eight-plates inserted/years	MAD Zone before eight-plate insertion	MAD Zone at present time with eight-plates in-stu
7	I	M	Right	Tibia	8.8	2.1	3	2
			Left	Tibia	8.8	2.1	4	2
8	I	M	Right	Tibia	8.6	0.3	4	3
			Left	Tibia	8.6	0.3	4	3
9	I	F	Right	Femur/tibia	9.1	0.7	3	0
			Left	Femur/tibia	9.1	0.7	3	0
10	II	M	Right	Tibia	13.0	1.1	3	2
			Left	Tibia	13.0	1.1	3	0

## Discussion

Knee deformity is common in children with MPS types I and II. Knee alignment in MPS has previously been described using the tibio-femoral angle only [8, 22]. Children with MPS have a high incidence of hip dysplasia [8] and therefore a normal tibio-femoral angle may not correlate with a normal mechanical axis. It is for this reason that MAD was used in this study.

In MPS I, a gradual increase in the mean MAD was seen in children of increasing age. This trend was less clear in children with MPS II. All MPS I children >8 years had valgus knee alignment. In MPS II children, 75 % >8 years had valgus knee alignment, 21 % had varus alignment and 4 % had neutral alignment. MPS I children tended to have greater knee deformity than MPS II children of the same age. No MPS II child had MAD through zone 4, although seven MPS I children did.

With increasing age, there was no change in the mLDFA in children with MPS I and MPS II. A valgus distal femur (mLDFA ≤ 85°) was more common in MPS I and a varus distal femur (mLDFA ≥ 91°) was more common in MPS II.

In MPS I children of increasing age, there was a steady increase in the mean MPTA. There was no clear trend in MPS II. All MPS I children >8 years had a valgus proximal tibia (mMPTA ≥ 91°). In MPS II children >8 years, 69 % had a valgus proximal tibia and 31 % had neutral alignment. No child of any age with MPS I or II had a varus proximal tibia (mMPTA ≤ 85°).

In individual children, the MAD worsened over time in approximately two-thirds of MPS I knees and half of MPS II knees. On average, it took 2 years for the MAD to progress one zone. In MPS I, the fastest deforming knee took 1 year to progress one MAD zone. In MPS II, the fastest deforming knee took <8 months to progress one MAD zone.

**Fig. 5:**
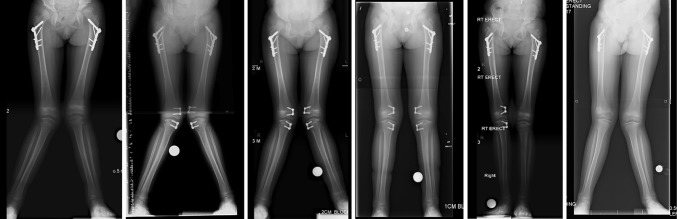
X-rays showing ‘typical’ improvement in knee alignment with insertion of eight-plates and recurrence of deformity following plate removal

Guided growth using eight-plates adequately corrected knee alignment in all cases. Recurrence of deformity was seen in three of four (75 %) cases at 1 year following plate removal. Correction was maintained in 1 case. This patient was skeletally mature at the time of eight-plate removal. X-rays of a ‘typical’ MPS I patient following eight-plate insertion and removal are shown in Fig. 5.

We believe there are 2 advantages to treating skeletally immature children with guided growth, despite deformity recurrence following plate removal. Firstly, deformity progression is delayed. The plates remain in situ for a mean of 1.6 years and it takes 1 year for deformity to recur following plate removal. Thus, there is no deformity progression for over 2 years. In this time period, deformity progression of over one MAD zone (based on our results) would be expected if the plates had not been inserted. Secondly, deformity is not allowed to reach a ‘critical’ level that could limit function. The correlation between knee deformity and function in children with MPS requires further research. Recurrent deformity can be treated by reapplication of the guided growth principles.

Knee deformity in children with MPS has previously been reported. Stoop et al. [7] described genu valgum in a series of 13 knees in 13 children with MPS I. They reported a high MPTA in 12/13 knees. A similar result was found in our series. Stoop et al. did not report overall knee alignment or the femoral contribution to knee deformity. They reported a maximum MPTA value between the ages of 3 and 5 years. This differs from our study where the mean MPTA continued to increase until at least 9 years of age.

Treatment with guided growth in MPS has previously been reported. Taylor et al. [22] reported 5 patients with MPS I treated with stapling of the medial tibial epiphysis. The tibio-femoral angle corrected from a mean of 17° to 10°. The contribution of the distal femur to the knee deformity and recurrence rates following staple removal are not reported.

Odunusi et al. [8] described treatment with guided growth in 8 valgus knees in children with MPS I using staples. There was complete correction of deformity in 2 knees and incomplete correction in 6 knees. Deformity recurred in 5 cases after staple removal and there was incomplete follow-up in 1 case. There was no recurrence in 2 knees. Both these patients were near skeletal maturity at the time of staple removal (age 14 and 15 years, respectively). This is the same as our study where there was no recurrence in one patient who had reached skeletal maturity prior to plate removal.

There are two possible strategies for treatment with guided growth. The first strategy involves performing surgery when knee alignment becomes ‘unacceptable’, e.g., when the MAD progresses to zone 3 on the assumption that gross deformity does alter gait mechanics and is associated with discomfort. Early treatment would correct the MAD and knee alignment would be maintained within specified limits to limit pain and maintain function. A disadvantage is that a child may require two or three surgical interventions during their childhood to achieve neutral limb alignment at skeletal maturity.

A second strategy would be to delay treatment with guided growth until the child is nearing skeletal maturity in an attempt to achieve normal knee alignment at the time skeletal maturity is reached. An advantage of this strategy is that it requires a single operation. A disadvantage is that a child may develop severe deformity that limits function; correction of a MAD in zone 4 may not occur so reliably as in a younger child particularly as the rate of deformity correction varies between individuals, so it would be difficult to estimate the time of plate insertion. If plates were inserted too early they would need to be removed with the risk of deformity recurrence. If the plates were inserted too late there would be under-correction of the deformity.

Our study has several strengths. It has a large study population for such a rare condition, there are several standing long-leg X-rays for many patients allowing disease progression to be reported over time and 3 important aspects of knee alignment have been reported (MAD, mLDFA and MPTA). This study also has weaknesses. It was performed retrospectively and is based solely on radiographic measurements that may not correlate to patient symptoms. Radiographs were taken at different ages and at different time intervals. Deformity progression was assessed in individual knees over a mean time period of 3 years. Studies with longer follow-up are necessary to assess progression throughout childhood. For the treatment arm of this study, only 8 knees underwent 1-year follow-up following plate removal, making this a case series rather than a true study. Nevertheless, we have shown that guided growth has the potential to improve significant deformity and maintain it in the short term.

Medical treatments for MPS I and MPS II [23, 24] have improved life expectancy and quality of life, resulting in more patients and doctors considering guided growth to correct knee alignment. When considering surgery, it is important to treat the source of the deformity (tibia, femur or both). Surgery should only be considered if the deformity is sufficiently severe or shows evidence of progression. This study shows that not all deformities progress in the medium-term. Patients should be aware of the high rate of deformity recurrence following plate removal and the need for further surgery in the future. It remains to be seen whether guided growth treatments can deliver a straight leg at skeletal maturity in children with MPS I and MPS II.
